# Dynamic Characterization of Antioxidant-Related, Non-Volatile, and Volatile Metabolite Profiles of Cherry Tomato During Ripening

**DOI:** 10.3390/antiox14111359

**Published:** 2025-11-13

**Authors:** Zhimiao Li, Sihui Guan, Rongqing Wang, Meiying Ruan, Qingjing Ye, Zhuping Yao, Chenxu Liu, Hongjian Wan, Guozhi Zhou, Yuan Cheng

**Affiliations:** 1 State Key Laboratory for Quality and Safety of Agro-Products, Vegetable Research Institute, Zhejiang Academy of Agricultural Sciences, Hangzhou 310021, China; 2 College of Agriculture, Shihezi University, Shihezi 832003, China

**Keywords:** cherry tomato, ripening, antioxidant, phenolic acids, flavonols, vitamins, volatilome, HPLC, UPLC-MS/MS, GC-MS

## Abstract

Cherry tomato is a notable dietary source of metabolites associated with antioxidant functions. However, how ripening reshapes primary, specialized, and volatile metabolites remains incompletely resolved. Green-ripe and red-ripe fruits were comparatively analyzed using targeted HPLC assays for quality indices and vitamins, UPLC–MS/MS for non-volatile metabolites, and HS-SPME–GC–MS for volatiles. Ripening was accompanied by a pronounced accumulation of lycopene and an increase in soluble solids, reflecting a shift of sugars toward glucose and fructose while sucrose remained low. Organic acids declined overall, with citric acid remaining predominant. The free-amino-acid pool expanded, with redistribution from GABA toward glutamate and aspartate. Vitamins exhibited stage-dependent patterns; antioxidant-related vitamins (A, E, and C) were higher at the red-ripe stage, indicating a compositional enhancement relevant to nutritional quality. Non-volatile metabolomics revealed 618 differentially accumulated metabolites, with phenolic acids, flavonoids, alkaloids, amino acids, and lipids as major classes. Phenolic acids and flavonols, dominated by hydroxycinnamoyl-quinic acids and quercetin/kaempferol glycosides, accumulated at the red-ripe stage, whereas steroidal glycoalkaloids decreased, suggesting conversion away from bitter or anti-nutritional constituents. GC–MS profiling identified 788 volatiles, with esters, terpenoids, and ketones contributing more than half of the volatilome. Ripening favored fruity–floral odorants such as β-ionone and (5Z)-octa-1,5-dien-3-one, while reducing green-leaf aldehydes. These stage-specific shifts in metabolite composition jointly define the sensory and nutritional maturation of cherry tomato. The identified metabolite markers provide a foundation for evaluating fruit maturity and guiding breeding toward improved quality attributes.

## 1. Introduction

Tomato (*Solanum lycopersicum* L.) is among the most widely consumed fruit vegetables worldwide and a model species for studying fleshy-fruit development [1]. Its market value and consumer acceptance are strongly determined by quality attributes arising from sugars, organic acids, amino acids, vitamins, and a diverse suite of volatile organic compounds [2,3]. Cherry tomato, a small-fruited cultivar group, has drawn particular attention because of its intense flavor and comparatively high soluble solids, which make it an informative system for dissecting the metabolic basis of fruit quality. Recent omics work further suggests that cherry tomatoes often accumulate higher levels of sugars, organic acids, and volatiles than large-fruited cultivars, reinforcing their utility for flavor research [4].

Fruit ripening is a coordinated physiological program that remodels color, texture, taste, and aroma through extensive metabolic reconfiguration. During the transition from the green-ripe to the red-ripe stage, tomato fruits typically exhibit sugar accumulation (glucose, fructose, sucrose), organic-acid rebalancing (notably citrate and malate), conversion within the free amino-acid pool, and the de novo synthesis of key aroma volatiles [5,6]. Tomato ripening proceeds from mature green through breaker, pink and light red to red ripe, each stage showing distinct metabolic profiles, with the green and red stages exhibiting the greatest differences [7]. From a sensory perspective, tomato flavor emerges from interactions between taste (sugars and acids) and aroma (volatiles) [8]. Although many volatiles have been detected, consumer liking is consistently explained by a smaller subset, and some volatiles can enhance perceived sweetness independently of sugar concentration [9]. Seminal chemical-genetic studies integrating tasting panels with metabolite and genomic data identified volatile constituents that contribute to perceived sweetness and overall liking, in some cases enhancing sweetness perception independently of sugar concentration [1,10]. This insight underscores why profiling both primary metabolites and volatiles is necessary to capture quality differences between ripening stages. Despite substantial progress, integrated stage-resolved datasets that connect primary metabolism, including sugars, organic acids, amino acids, and vitamins, to the volatilome remain relatively scarce, especially in cherry tomato. Recent syntheses emphasize the need for quantitative links between these layers to understand ripening-associated shifts and to inform breeding for improved flavor [11,12].

Addressing these gaps, the present study undertakes a comparative analysis of fruit quality traits and metabolite profiles in cherry tomato at the green-ripe and red-ripe stages. In this research, sugars, organic acids, and amino acids were quantified using targeted assays, while primary and volatile metabolites were profiled through complementary metabolomic platforms. By integrating quality indices with metabolite profiling and pathway annotation, we quantify compositional shifts between the green and red stages, identify volatile classes most responsive to ripening, and relate primary-metabolite variation to volatile profiles to define the metabolic drivers of quality. Beyond providing a data-driven description of stage-specific metabolic states, these results are expected to inform breeding targets and postharvest decisions aligned with consumer-oriented flavor enhancement.

## 2. Materials and Methods

### 2.1. Plant Material

Cherry tomato cultivar ‘Zheyingfen No. 1’ was cultivated in the spring of 2024 at the Yangdu experimental greenhouse station of the Zhejiang Academy of Agricultural Sciences, Zhejiang Province, China (30°27′ N, 120°2′ E). Plants were grown under standard horticultural practices in a greenhouse environment, with a spacing of 40 cm between plants and 50 cm between rows. Routine water and fertilizer management followed local commercial cultivation guidelines. Fruits representing two developmental stages were collected: 35 days post anthesis (green-ripe stage, GRS) and 45 days post anthesis (red-ripe stage, RRS). For each treatment, 12 plants were used, and every four plants were grouped as one biological replicate. A total of three independent replicates were established for each treatment.

### 2.2. Measurement of Quality Indicators

Quantitative analyses of sugars, organic acids, amino acids, γ-aminobutyric acid (GABA), and vitamins were conducted following standardized chromatographic procedures. Except for vitamin C, which was extracted according to a published method [13] all assays were performed using validated Standard Operating Procedures (SOPs) established by Suzhou Grace Biotechnology Co., Ltd. (Suzhou, China). Analytical protocols were harmonized to include three consistent stages: sample extraction, chromatographic separation, and quantification.

Soluble sugars: Approximately 0.2 g of homogenized fruit powder was extracted with 1 mL of 40% acetonitrile by ultrasonication for 30 min at room temperature. The extract was centrifuged (12,000 rpm, 10 min) and filtered through a 0.22 µm membrane. Sugars were analyzed using an LC-100 HPLC system equipped with a Dikma Polyamino HILIC column (250 × 4.6 mm, 5 µm) and a Shodex RI-201H refractive index detector. The mobile phase consisted of acetonitrile (A) and water (B) = 60:40 (*v*/*v*) under isocratic elution, with a flow rate of 1.0 mL min^−1^, column temperature 40 °C, and injection volume 10 μL. Fructose, glucose, and sucrose were quantified by external calibration (R^2^ > 0.98).

Organic acids: For organic acids, 0.2 g of sample was extracted with 1 mL ultrapure water, sonicated for 30 min, and centrifuged (12,000 rpm, 4 °C, 10 min). The supernatant was filtered before injection. Chromatographic separation was performed on a Shimadzu LC-20AT system with a C18 reversed-phase column (150 × 4.6 mm, 5 µm). The mobile phase consisted of methanol (A) and 0.2% NaH_2_PO_4_ (B, pH 2.7) = 3:97 (*v*/*v*). The flow rate was 0.6 mL min^−1^, column temperature 30 °C, and detection wavelength 210 nm.

γ-Aminobutyric acid (GABA): Approximately 0.2 g of sample was extracted with 1.5 mL water and digested overnight. After ultrasonication (40 min) and centrifugation (12,000 rpm, 10 min), the supernatant was derivatized with 2.0 M triethylamine and 0.2 M phenyl isothiocyanate (PITC), reacted for 30 min at room temperature, extracted with n-hexane, and filtered (0.22 µm). HPLC analysis was carried out using a Shimadzu LC-20A system with a C18 column (250 × 4.6 mm, 5 µm). The mobile phases were A: 0.1 M sodium acetate solution and B: acetonitrile/water, under gradient elution. The flow rate was 1 mL min^−1^, column temperature 40 °C, and UV detection at 254 nm.

Vitamin C: Extraction followed Sánchez-Moreno et al. [13], using 3% metaphosphoric acid as the extraction solvent to prevent oxidation. After ultrasonication (30 min) and centrifugation (12,000 rpm, 10 min), the supernatant was filtered for analysis. Chromatography was performed on a Shimadzu LC-20AT system with a C18 column (250 × 4.6 mm, 5 μm). The mobile phase consisted of methanol (A) and 0.02 M KH_2_PO_4_ (B) = 10:90 (*v*/*v*), flow rate 1 mL min^−1^, column temperature 35 °C, and detection at 245 nm.

Vitamins A and E: About 0.2 g of homogenized tissue was extracted with 1 mL of 1% BHT in ethanol and 1 mL of 10% KOH at 85 °C for 40 min. The mixture was extracted with 2 mL petroleum ether, washed, evaporated to dryness, and redissolved in methanol. HPLC conditions: Shimadzu LC-20A, C18 column, mobile phase methanol/water = 85:15, flow rate 1 mL min^−1^, column temperature 30 °C, and detection at 325 nm.

Vitamins B_1_, B_2_, and B_6_: Samples were extracted with ultrapure water by ultrasonication (40 min), centrifuged, and filtered (0.22 µm). Analysis was performed on a Shimadzu LC-20A with a C18 column (250 × 4.6 mm, 5 µm). The mobile phase was acetonitrile (A)/0.01% phosphoric acid (B) = 15:85 (*v*/*v*), flow rate 0.8 mL min^−1^, column temperature 30 °C, and UV detection at 270 nm.

Total free amino acids (colorimetric determination): Total free amino acids were determined using a ninhydrin colorimetric assay in accordance with the manufacturer’s SOP (Suzhou Grace Biotechnology Co., Ltd., Suzhou, China). Briefly, 0.2 g of homogenized fruit tissue was extracted according to the kit instructions. Under acidic conditions, amino acids react with ninhydrin to form a blue–purple diketohydrindylidene–diketohydrindamine complex exhibiting a characteristic absorption peak at 570 nm. Absorbance was measured using a multifunctional microplate reader (MD-M2, Molecular Devices, San Jose, CA, USA), and total free amino acid content (µmol g^−1^ FW) was calculated following the manufacturer’s formula. This assay quantifies the overall pool of free amino acids and does not distinguish individual species.

Individual amino acids (HPLC analysis): Individual amino acids were profiled using pre-column derivatization HPLC analysis based on validated SOPs of Suzhou Grace Biotechnology Co., Ltd. Approximately 0.2 g of ground sample was extracted with 1.5 mL of 70% ethanol, ultrasonicated for 30 min, centrifuged (12,000 rpm, 10 min), evaporated, and redissolved in 1 mL of ultrapure water. Samples were derivatized with phenyl isothiocyanate (PITC) prior to analysis. Chromatographic separation was carried out on a Wufeng LC-100 HPLC system equipped with an amino acid analysis column (250 × 4.6 mm, 5 µm). The mobile phases were A: 0.05 M sodium acetate (pH 6.5) and B: methanol/acetonitrile/water = 20:60:20 (*v*/*v*/*v*). A linear gradient was applied at a flow rate of 1.0 mL min^−1^, column temperature 35 °C, and detection at 254 nm. Seventeen amino acids were quantified using external calibration with authentic standards (R^2^ > 0.99). This method allows differentiation of individual amino acid species and complements the total amino acid assay.

Soluble solids content was measured following the SOP of Suzhou Grace Biotechnology Co., Ltd. using a refractometric method. After homogenization and extraction, the clear supernatant was analyzed using a digital refractometer, and results were expressed as percentage (%), representing the total soluble sugar level and fruit maturity.

Lycopene content: Approximately 0.2 g of homogenized tomato tissue was ground in a mortar and extracted with 3 mL of ethanol. The mixture was vortexed for 5 min and sonicated for 30 min at 25 °C, followed by centrifugation at 12,000× *g* for 10 min. The precipitate was re-extracted with 3 mL of ethyl acetate under the same conditions. The two supernatants were combined, evaporated to dryness under nitrogen, and re-dissolved in 2 mL of ethyl acetate. The resulting extract was filtered through a 0.22 µm syringe filter into an amber vial for HPLC analysis. Chromatographic analysis was carried out on a Shimadzu LC-20A HPLC system equipped with a C18 column (250 × 4.6 mm, 5 µm) and a photodiode array (PDA) detector. The mobile phase consisted of methanol (A) and acetonitrile: ethyl acetate = 50: 50 (B), mixed in a 1:1 ratio (A: B = 50:50, *v*/*v*) under isocratic elution. The flow rate was 1.0 mL min^−1^, column temperature 30 °C, and detection wavelength 472 nm. The injection volume was 10 µL.

### 2.3. UPLC-MS/MS Analysis

#### 2.3.1. Dry Sample Extraction

Freeze-dried tomato samples were used for metabolite extraction. Samples were lyophilized for 63 h using a Scientz-100F freeze-dryer and ground into fine powder with a Retsch MM400 grinder at 30 Hz for 1.5 min. Approximately 50 mg of the powdered sample was accurately weighed using an MS105Dμ analytical balance and extracted with 1.2 mL of pre-cooled 70% methanol (−20 °C) containing the internal standard 2-chlorophenylalanine (1 mg L^−1^, purity 98%, Macklin, Shanghai, China). The extraction mixture was vortexed for 30 s every 30 min, repeated six times to ensure sufficient extraction efficiency. After centrifugation at 12,000 rpm for 3 min, the supernatant was collected and filtered through a 0.22 µm microporous membrane. The filtrate was transferred into autosampler vials for subsequent UPLC–MS/MS analysis.

#### 2.3.2. UPLC Conditions

Sample extracts were analyzed using a UPLC–ESI–MS/MS system consisting of an ExionLC™ AD UPLC and a QTRAP 6500 mass spectrometer (SCIEX, Framingham, MA, USA). Chromatographic separation was performed on an Agilent SB-C18 column (1.8 µm, 2.1 mm × 100 mm). The mobile phase consisted of solvent A (water with 0.1% formic acid) and solvent B (acetonitrile with 0.1% formic acid). The gradient program started at 95% A/5% B, linearly shifted to 5% A/95% B within 9 min, held for 1 min, then returned to 95% A/5% B within 1.1 min and maintained for 2.9 min. The flow rate was 0.35 mL/min, the column oven was maintained at 40 °C, and the injection volume was 2 µL. The eluent was introduced into an ESI triple quadrupole–linear ion trap mass spectrometer (QTRAP-MS) for detection.

#### 2.3.3. ESI-Q TRAP-MS/MS

The ESI source parameters were set as follows: source temperature, 500 °C; ion spray voltage, +5500 V in positive mode and –4500 V in negative mode; ion source gases I and II at 50 and 60 psi, respectively; curtain gas at 25 psi; and collision-activated dissociation (CAD) at a high level. Multiple reaction monitoring (MRM) mode was used for QQQ scans with nitrogen as the collision gas at medium setting. Declustering potential (DP) and collision energy (CE) for each transition were optimized individually. Specific MRM transitions were monitored for defined retention-time periods according to the elution characteristics of the metabolites.

### 2.4. GC-MS Analysis

#### 2.4.1. Solid/Liquid Samples Class II

Plant materials were harvested, weighed, immediately frozen in liquid nitrogen, and stored at –80 °C until analysis. Samples were ground into fine powder under liquid nitrogen, and 500 mg (≈1 mL) of powder was transferred to a 20 mL headspace vial (Agilent, Palo Alto, CA, USA) containing a saturated NaCl solution to suppress enzymatic activity. The vials were sealed with crimp-top caps equipped with TFE–silicone septa (Agilent). For SPME analysis, each vial was equilibrated at 60 °C for 5 min, after which a 120 µm DVB/CWR/PDMS fiber (Agilent) was exposed to the headspace for 15 min at 60 °C.

#### 2.4.2. GC-MS Conditions

After sampling, VOCs were thermally desorbed from the SPME fiber in the GC injection port (Agilent 8890) at 250 °C for 5 min under splitless mode. Separation and detection were performed on an Agilent 8890 GC coupled to a 7000D mass spectrometer, using a DB-5MS capillary column (30 m × 0.25 mm × 0.25 µm, 5% phenyl-polymethylsiloxane). Helium served as the carrier gas at a constant flow of 1.2 mL/min. The injector temperature was maintained at 250 °C. The oven program was as follows: initial 40 °C (held 3.5 min), ramped to 100 °C at 10 °C/min, to 180 °C at 7 °C/min, and to 280 °C at 25 °C/min, then held for 5 min. Mass spectra were acquired in electron impact (EI) mode at 70 eV. The quadrupole, ion source, and transfer line temperatures were set at 150, 230, and 280 °C, respectively. Data were collected in selected ion monitoring (SIM) mode for the identification and quantification of analytes.

### 2.5. Statistical Analysis

The relative odor activity value (rOAV) is a threshold-based approach used to identify key aroma-active compounds in horticultural crops [14]. It reflects the contribution of each VOC to the overall aroma profile of a sample. In this study, the compound with the highest rOAV in tomato fruits was set to 100 as the reference. Compounds with rOAV values ≥ 1 were considered key aroma contributors, while those with 0.1 < rOAV < 1 were classified as aroma modifiers [15]. rOAVᵢ = Cᵢ/Tᵢ, where rOAVᵢ represents the relative odor activity value of compound i, Cᵢ is the relative concentration of the compound (µg/g or µg/mL), and Tᵢ is the odor threshold of the compound (µg/g or µg/mL) [16,17].

Data processing and compilation were performed using Microsoft Excel 2021 (Microsoft Corporation, Redmond, WA, USA). Statistical analyses and bar graph visualizations were conducted using GraphPad Prism version 10.0 (GraphPad Software, San Diego, CA, USA). One-way analysis of variance (ANOVA) was carried out in SPSS to evaluate the significance of differences among treatments. Heatmaps, Venn diagrams, and related visualizations were generated using the online platform available at https://cloud.metware.cn (accessed on 1 January 2025). Least significant difference tests (Student’s *t*-test) were conducted to analyze the differences between treatments (*p* < 0.05).

## 3. Results and Discussion

### 3.1. Effects of Ripe Stages on Nutritional Quality of Cherry Tomato Fruits

**Figure 1:**
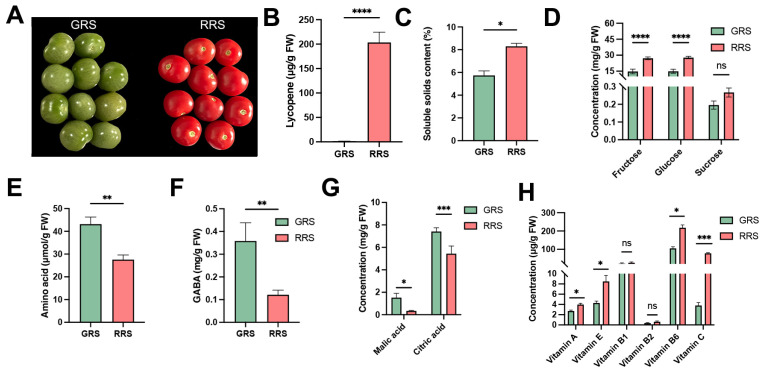
Ripening-associated changes in phenotype and nutritional quality of cherry tomato fruits. (**A**) Representative fruits at the GRS and RRS. (**B**) Lycopene content. (**C**) Soluble solids content. (**D**) Soluble sugars (fructose, glucose, sucrose; a *y*-axis break is used to display sucrose). (**E**) Total amino acids. (**F**) GABA. (**G**) Organic acids: malic acid and citric acid. (**H**) Vitamins A, E, B1, B2, B6, and C. Bars show mean ± SD; significance is indicated as *p* < 0.05 (*), *p* < 0.01 (**), *p* < 0.001 (***), *p* < 0.0001 (****); ns, not significant. GRS, green-ripe stage; RRS, red-ripe stage. Values are expressed as means ± SE of at least 3 replicates.

Fruit ripening is accompanied by a series of biochemical and physiological changes that shape both nutritional quality and flavor. Key indices such as soluble sugars, organic acids, amino acids, vitamins, and pigments are tightly associated with the transition from immature green to fully ripe red stages, providing an integrated reflection of fruit development and consumer preference. To investigate these relationships, we compared cherry tomato fruits at the GRS and the RRS. At the phenotypic level, fruits exhibited a clear color transition from green to red during ripening (Figure 1A), which was accompanied by a dramatic accumulation of lycopene (Figure 1B). SSC also significantly increased at the red-ripe stage compared with the green-ripe stage (Figure 1C), consistent with previous reports that sugar accumulation is a hallmark of tomato ripening [18]. Sugar profiling revealed that both fructose and glucose contents markedly increased from the GRS to the RRS, while sucrose remained at a low level without significant changes (Figure 1D). This agrees with earlier studies indicating that sucrose is rapidly hydrolyzed in tomato fruits, leaving glucose and fructose as the dominant soluble sugars [19].

Total free amino acids, measured by the ninhydrin colorimetric method, showed a decrease from GRS to RRS (Figure 1E), mainly due to the sharp decline of GABA (Figure 1F). To further characterize compositional variation, individual amino acids were quantified using HPLC (Table 1). Unlike the colorimetric assay that determines total free amino acids, HPLC provides compound-specific concentrations, allowing differentiation among amino acid types. Although the overall amino acid pool decreased, the concentrations of several key amino acids—including Glu, Asp, and Ser—increased significantly at the red-ripe stage, indicating metabolic redistribution rather than uniform depletion. Additional increases were observed for Ala, His, Pro, Met, and Arg, while Val and Ile decreased, and others showed no significant change. Meanwhile, GABA, quantified separately, declined sharply at RRS, consistent with previous findings that its accumulation peaks at the green-ripe stage and drops during ripening due to reduced glutamate decarboxylase activity [20]. Collectively, these results demonstrate that ripening involves a reallocation of nitrogen metabolism from GABA enrichment in green fruits toward Glu and Asp accumulation in ripe fruits, contributing to the umami characteristics of cherry tomato [21,22].

Among organic acids, malic acid exhibited a sharp reduction, whereas citric acid, although also decreasing, remained the predominant organic acid at both stages (Figure 1G). These results confirm earlier reports that ripening is associated with a decline in acidity, particularly due to the degradation of malic acid, leading to a higher sugar/acid ratio and improved flavor [23]. Recent syntheses similarly note that organic acids serve as respiratory substrates and generally decline during tomato ripening, and citric and malic acids together account for the vast majority of the acid pool, with citric acid remaining dominant [24]. Vitamin analysis showed diverse trends (Figure 1H). Vitamins A, E, and C increased during ripening, consistent with studies showing carotenoid and α-tocopherol accumulation in red-ripe fruit and with reviews highlighting the up-regulation/central role of ascorbic acid pathways during maturation [25]. Vitamin B6 also showed a significant increase at the red-ripe stage, indicating its potential involvement in antioxidant defense and amino-acid metabolism during fruit maturation. In contrast, vitamins B1 and B2 generally exhibited small or cultivar-dependent changes across ripening. Notably, despite the decline in the global amino-acid pool, the targeted HPLC data indicate selective enrichment of flavor-relevant amino acids at RRS, pointing to compositional reprogramming rather than uniform depletion. This pattern aligns with a ripening-associated downshift of the GABA shunt and a redirection of carbon–nitrogen flux toward Glu/Asp via transamination/GDH-linked reactions [11,26]. From an applied standpoint, Glu/Asp enrichment together with GABA depletion could serve as metabolic markers for harvest timing and for breeding programs targeting enhanced umami in cherry tomato.

### 3.2. Effects of Different Ripe Stages on Non-Volatile Substances in Tomato Fruits

#### 3.2.1. Comparative Analysis of the Composition and Content of Primary Metabolic Products in Tomato Fruits at Different Ripe Stages

**Figure 2:**
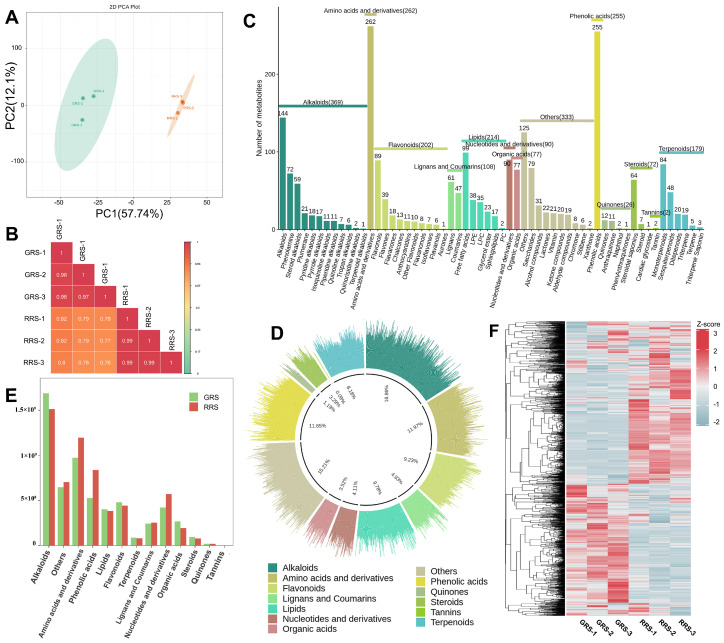
Composition analysis of different ripe stages on non-volatile substances in tomato fruits. (**A**) PCA analysis of sample data in each group. (**B**) Pearson product-moment correlation coefficients; (**C**) The relative content histogram of primary metabolites in each group. (**D**) The relative content histogram of primary metabolites in each group. (**E**) The proportion of primary metabolites in each category. (**F**) The heatmap of primary metabolite content in each group of samples.

To capture global remodeling of the non-volatile metabolome during ripening, we performed untargeted UPLC-MS/MS metabolomics on cherry tomato at the GRS and RRS stages. The dataset provides a stage-resolved view of non-volatile metabolite profiles and enables comparisons at both feature and class levels. Principal component analysis (PCA) revealed a clear separation between GRS and RRS samples, with PC1 and PC2 explaining 57.74% and 12.10% of the total variance, respectively (Figure 2A). Biological replicates clustered tightly within each stage, indicating high data reproducibility (Figure 2B). To further validate stage discrimination, partial least squares discriminant analysis (PLS-DA) was conducted. The PLS-DA model was cross-validated and assessed by permutation testing (R^2^Y = 0.996, Q^2^ = 0.943), confirming its robustness and the absence of overfitting (Figure S1). The identified metabolites encompassed 13 categories, namely 369 alkaloids (16.86%), 262 amino acids and derivatives (AAS, 11.97%), 255 phenolic acids (11.65%), 214 lipids (9.78%), 202 flavonoids (9.23%), 179 terpenoids (8.18%), 108 lignans and coumarins (4.93%), 90 nucleotides and derivatives (4.11%), 77 organic acids (3.52%), 72 steroids (3.29%), 26 quinones (1.19%), 2 tannins (0.09%) and 333 other compounds (15.21%) (Figure 2C,D, Table S1). Furthermore, out of the 214 lipids identified, they can be categorized into six subclasses: free fatty acids, lysophosphatidylethanolamine (LPE), lysophosphatidylcholine (LPC), glycerol ester, sphingolipids, and phosphatidylcholine (PC), comprising 99, 38, 35, 23, 17, and 2 compounds, respectively. Each subclass of lipids serves distinct functions. The relative content of the primary metabolites obtained by the detection was analyzed (Figure 2E). It was found that the total content of the primary metabolites in the two tomato fruits samples showed the following trend: RRS > GRS. The effect of RRS on the content of tomato metabolites was more obvious than that of GRS, and the total content of amino acids and derivatives, phenolic acids, and nucleotides and derivatives increased significantly. Through the heatmap analysis of all the primary metabolites obtained by identification (Figure 2F), it can be seen from the results that there were significant differences in the content of primary metabolites in different ripe samples. Unsupervised hierarchical clustering resolved two major modules with reciprocal stage biases, one enriched in GRS samples and the other in RRS samples, together with several smaller clusters that likely reflect pathway-specific shifts during ripening.

#### 3.2.2. The Influence of Different Maturity Stages on the Composition and Content of Primary Metabolites in Tomato Fruits

**Figure 3:**
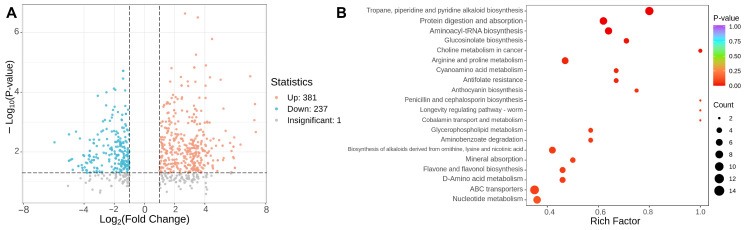
Analysis of differential metabolites in tomato fruits with different ripe stages. (**A**) Each point in the volcano map represents a metabolite. The blue point represents a downregulated differential metabolite, and the orange point represents an upregulated differential metabolite. (**B**) Enrichment bubble plot by KEGG. Rich factor values approaching 1.0 denote a high proportion of matched metabolites within a pathway, and warmer colors represent lower adjusted *p* values, confirming strong statistical support. Larger bubbles reflect pathways contributing more matched features.

In order to gain a deeper insight into the alterations of primary metabolites in tomato fruits subjected to different ripe stages, this study employed the criteria of VIP > 1, fold change ≥ 2 or ≤ 0.5 to identify the distinct metabolites between GRS and RRS samples (Figure 3A, Table S3). The findings revealed variations in the number of unique metabolites identified in the RRS versus GRS comparison sets, totaling 618 (381 upregulated and 237 downregulated) (*p*-value ≤ 0.05). To identify the key metabolic pathways of differential accumulated metabolites (DAMs) during tomato ripe stages, Kyoto Encyclopedia of Genes and Genomes (KEGG) enrichment analysis was conducted and the top twenty significant metabolite pathways were shown in Figure 3B. Bubble-plot analysis highlighted multiple significantly enriched pathways across the two ripening stages. Among primary-metabolism routes, enrichment was observed for glycerophospholipid metabolism, aminoacyl-tRNA biosynthesis, arginine and proline metabolism, D-amino acid metabolism, cyanoamino acid metabolism, and nucleotide metabolism, indicating broad adjustments in lipid remodeling and nitrogen metabolism. In the specialized-metabolism tier, several phenylpropanoid-related branches and nitrogenous secondary pathways were over-represented, including flavone and flavonol biosynthesis, anthocyanin biosynthesis, tropane/piperidine/pyridine alkaloid biosynthesis, biosynthesis of alkaloids derived from ornithine, lysine and nicotinic acid, and glucosinolate biosynthesis, consistent with ripening-associated reprogramming of defense and color-associated metabolites. Additional terms such as ABC transporters, protein digestion and absorption, mineral absorption, and aminobenzoate degradation suggest changes in transport capacity and cofactor turnover that accompany the metabolic shift. Notably, alkaloid biosynthesis and aminoacyl-tRNA biosynthesis showed both large bubble sizes and low *p* values, whereas flavone and flavonol biosynthesis and glycerophospholipid metabolism combined high Rich factors with clear stage bias. Taken together, the enriched pathways span lipids, amino acids and derivatives, phenolic acids and flavonoids, as well as alkaloids, which are the five compound classes most relevant to ripening differences. Downstream analyses therefore focus on these classes to resolve their stage-dependent trajectories and contributions to quality.

#### 3.2.3. Phenolic Acids

**Figure 4:**
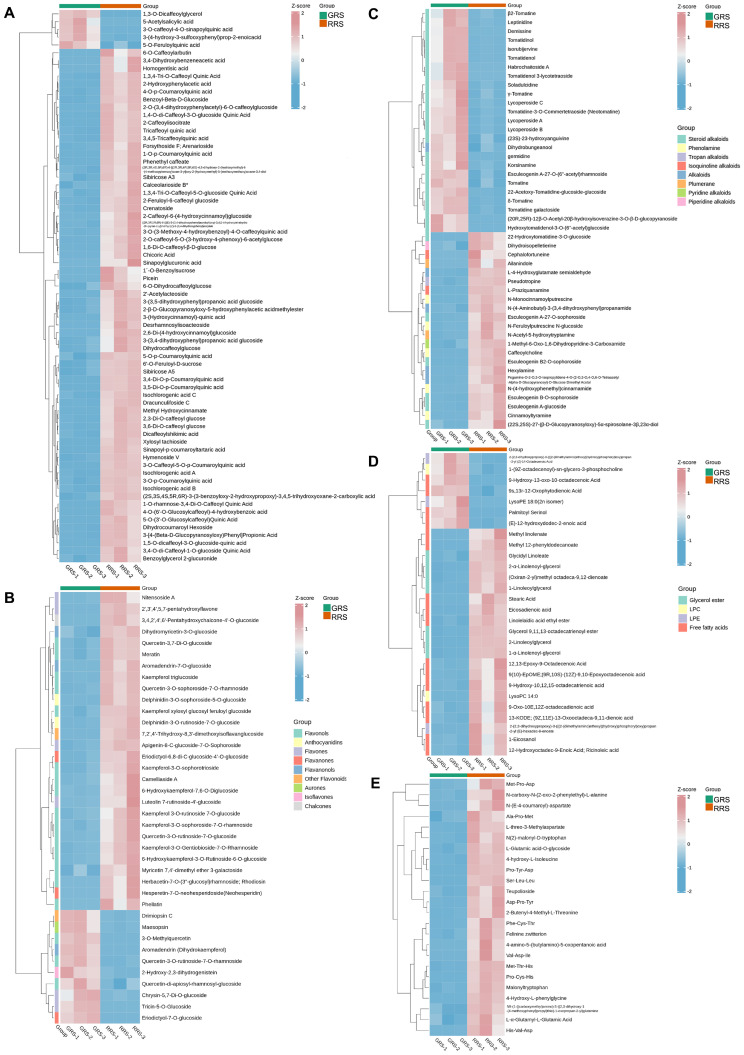
Heatmaps of lipids (**A**), phenolic acids (**B**), flavonoids (**C**), alkaloids (**D**), lipids and amino-acids and derivatives (**E**) in DAMs.

Phenolic acids, a major class of non-flavonoid polyphenols, confer health benefits owing to their antioxidant activity [27]. In this study, across the GRS and RRS of tomato, alkaloids and amino acids and derivatives were the two most abundant non-volatile metabolite classes, while phenolic acids ranked third but represented the most compositionally diverse and significantly altered secondary-metabolite group (Figure 4A). The class-resolved heat map revealed a pronounced stage bias: most phenolic acids displayed higher standardized intensities at the RRS than at the GRS, forming a large RRS-enriched module with only a smaller subset retaining GRS preference. This pattern indicates that ripening favors both the quantitative build-up and the qualitative diversification of phenolic acids rather than a uniform increase in a few individual compounds. Compositionally, the profile was dominated by hydroxycinnamoyl–quinic acid esters, that is, conjugates of quinic acid with caffeic acid, ferulic acid, and p-coumaric acid. Representative members included caffeoylquinic acid isomers (commonly referred to as chlorogenic and neochlorogenic acids), feruloylquinic acid, and p-coumaroylquinic acid. These conjugates accounted for the majority of the RRS-enriched signals, consistent with the expected activation of phenylpropanoid metabolism during the transition to full ripeness. The KEGG over-representation analysis supported this interpretation by listing phenylpropanoid biosynthesis among the top enriched pathways in the stage comparisons, together with ancillary routes that provide precursors and cofactors for phenylpropanoid assembly. From a functional perspective, the preferential accumulation of hydroxycinnamoyl-quinates is noteworthy because these molecules are widely associated with antioxidant capacity and with beneficial effects on lipid and energy metabolism reported in nutritional studies [28]. Literature has linked chlorogenic-acid family members to reduced oxidative damage and improved metabolic markers, and neochlorogenic-acid isomers have been reported to lessen cellular lipid accumulation [29,30,31]. We observed a broad increase in phenolic acid conjugates at the RRS relative to the GRS, with hydroxycinnamoyl-quinic acid esters (caffeoyl-, feruloyl-, and p-coumaroyl-quinates) showing the clearest stage bias. This RRS-oriented buildup is consistent with reports that plant developmental progression elevates phenolic acids not only in *Toona sinensis* microgreens but also in other species, reflecting a conserved metabolic trend during maturation [32]. Taken together, the data support a model in which ripening coordinately strengthens the phenylpropanoid branch centered on hydroxycinnamoyl-quinic esters, providing a mechanistic basis for the class-level enrichment seen in the heat map and yielding candidate markers for stage discrimination and quality assessment.

#### 3.2.4. Flavonoids

Flavonoids are multifunctional plant metabolites that provide antioxidant and UV-screening capacity, contribute to defense and pigmentation, and can modulate bitterness/astringency in foods [33]. As illustrated in Figure 4B, the tomato flavonoid biosynthetic pathway encompasses multiple subclasses, including flavonols, flavones, flavanones, flavanonols, aurones, isoflavones, chalcones, and a few anthocyanidin intermediates that are typically inactive in red-fruited cultivars. Most compounds occurred as O-glycosides, typically 3-O glycosides for flavonols (quercetin/kaempferol cores) and 7-O glycosides for flavones (luteolin/apigenin cores). From the GRS to the RRS, a general upward shift in flavonoid abundance was observed: flavones and the majority of quercetin/kaempferol glycosides increased toward the RRS, whereas a small subset of flavones and flavonols decreased or remained unchanged. Despite these exceptions, most differentially accumulated flavonoids reached higher levels at RRS, indicating a ripening-associated reinforcement of the flavonoid branch. Flavonols were the predominant constituent at the RRS, accompanied by frequent detection of kaempferol, including 6-hydroxykaempferol-3-O-rutinoside-6-O-glucoside, 6-hydroxykaempferol-7,6-O-diglucoside, kaempferol-3-O-sophoroside-7-O-rhamnoside, kaempferol triglucoside, kaempferol-3-O-sophorotrioside, Kaempferol xylosyl glucosyl feruloyl glucoside, kaempferol-3-O-Gentiobioside-7-O-Rhamnoside, and kaempferol 3-O-rutinoside 7-O-glucoside. Quercetin and kaempferol derivatives, such as quercetin-3-O-rutinoside-7-O-glucoside, quercetin-3,7-di-O-glucoside, and kaempferol-3-O-rutinoside, were also detected and showed higher accumulation at the RRS. Both compounds are well-recognized flavonols with strong antioxidant properties, contributing to the enhanced antioxidant potential of red-ripe fruits [34,35]. This compositional pattern is consistent with activation of the phenylpropanoid to flavonoid pathway during ripening, in which metabolic flux from p-coumaroyl-CoA through CHS, CHI, and F3H and then through FLS and F3′H favors the build-up of flavonol 3-O-glycosides at later stages. In agreement, KEGG enrichment recovered flavone and flavonol biosynthesis among the top stage-discriminating pathways. In sum, these results suggest that cherry tomato, especially when harvested at the RRS, is a more valuable source of natural dietary flavonoids.

#### 3.2.5. Alkaloids

Tomato steroidal glycoalkaloids, such as α-tomatine, play crucial roles in plant defense against pathogens and herbivores, and also exhibit pharmacological properties including antimicrobial, anti-inflammatory, and antioxidant activities in humans [36,37]. In this study, the identified alkaloids encompassed 8 categories, namely 30 steroid alkaloids, 5 alkaloids, 5 phenolamine, 2 isoquinoline alkaloids, 2 plumerane, 1 tropan alkaloids, 1 pyridine alkaloids, and 1 piperidine alkaloids (Figure 4C). Previous studies have demonstrated that α-tomatine, and likely δ-tomatine, accumulate to high levels in green (immature) tomato fruits and decline markedly during ripening [36,38]. In this study, we found that for most of the tomatidenols, such as δ-tomatine, tomatidine-3-O-commertetraoside (neotomatine), γ-tomatine, and β2-tomatine, the levels in RRS were significantly lower than those in GRS. This decrease is mainly attributed to the enzymatic conversion of glycoalkaloids into less toxic derivatives, particularly esculeoside A, and further into its aglycone esculeogenin A, thereby reducing bitterness and toxicity and ultimately improving the sensory quality of ripe fruit [39]. From an ecological perspective, this developmental shift reflects a balance between defense and reproduction: in the GRS, elevated glycoalkaloid concentrations function as chemical defenses against pathogens and herbivores to protect developing tissues, whereas their reduction in the RRS minimizes antinutritional properties and facilitates seed dispersal by frugivores [40]. Together, these findings suggest that the dynamic regulation of steroidal alkaloids is closely linked to both plant defense strategies and ecological adaptation.

#### 3.2.6. Lipids

Lipids underpin membrane architecture, energy storage, and signaling during fruit maturation [41,42]. During tomato maturity, the major components of lipids altered significantly, consisting of 16 free fatty acids (FFAs), 7 glycerol esters, 3 lyso-phosphatidylethanolamine (LPE) and 2 lyso-phosphatidylcholine (LPC) (Figure 4D). Free fatty acids, especially the short chain species acetate, propionate, and butyrate, are associated with beneficial effects on human metabolic, immune, and gut health [43]. The heat map revealed a clear stage bias: a large module comprising FFAs and several glycerol ester was elevated at RRS, whereas a subset of LPC/LPE showed flat or modestly decreasing trends from GRS to RRS. Class-level summaries corroborated these patterns, with total lipid intensity higher at RRS than at GRS. At the species level, RRS showed a clear shift toward unsaturated acyl pools. Monoacylglycerols bearing linoleoyl or α-linolenoyl chains were prominent at RRS, including 1/2-linoleoylglycerol, 1/2-α-linolenoyl-glycerol, and glycerol 9,11,13-octadecatrienoyl ester. Consistent derivatives such as methyl linolenate and linolelaidic acid ethyl ester were detected, together with the longer-chain unsaturated fatty acid eicosadienoic acid. A second hallmark of RRS was the broader presence of C18-derived oxylipins, including 9-hydroxy-10,12,15-octadecatrienoic acid, 12,13-epoxy-9-octadecenoic acid, 13-KODE; (9Z,11E)-13-oxooctadeca-9,11-dienoic acid, and 9-oxo-10E,12Z-octadecadienoic acid. In contrast, two LPE showed modestly decreasing trends from GRS to RRS, including 2-(2,3-dihydroxypropoxy)-3-(((2-(dimethylamino)ethoxy)(hydroxy) phosphoryl)oxy)propan-2-yl (Z)-14-octadecenoic acid, and LysoPE 18:0 (2n isomer). LPE has been implicated as a defense-promoting signal during early plant development. The modest decline in phospholipids during tomato ripening is likely attributable to stage-dependent regulation of glycerophospholipid catabolism, including phospholipase-mediated deacylation and subsequent turnover within the Lands cycle [44]. These patterns are consistent with lipid remodeling known to accompany tomato ripening [45]. Parallel reports show changes in membrane lipid classes and lipid-modifying enzyme activities as fruit ripens, supporting a model in which unsaturated acyl pools expand and oxygenated derivatives diversify as chloroplasts convert to chromoplasts and aroma precursors shift from early C6 aldehydes toward later pathways [42,46]. These species-level signatures align with established ripening biology and provide tractable markers for stage discrimination and for linking non-volatile lipid status to pigment accumulation and aroma development [41,47].

#### 3.2.7. Amino-Acids and Derivatives

As shown in Figure 4E, tomato was abundant in small molecule peptides, in addition to having a comprehensive amino acid composition. In this study, a total of 24 differential amino acids and their derivatives were identified, and notably, all metabolites showed significantly higher abundance in the RRS stage compared with the GRS stage. Tripeptides, as natural small peptides, possess multiple functions including antioxidant, anti-inflammatory, metabolic regulation, improvement of skin health, and potential antihypertensive activity, and thus have important application value in both plant and human health [48,49]. The tripeptides identified in this study, such as Met-Thr-His, Met-Pro-Asp, Pro-Tyr-Asp, Phe-Cys-Thr, and Asp-Pro-Tyr, exhibited higher abundance in the RRS stage, reflecting a dynamic transition from early accumulation to subsequent degradation and utilization. In addition, some amino acid derivatives, such as malonyltryptophan and teupolioside, accumulated at higher levels in the RRS stage, suggesting that they may serve as intermediates in the transformation of aromatic metabolism. Derivatives with special side chains, such as 2-butenyl-4-methyl-L-threonine, displayed large fluctuations between the two stages, which may be associated with metabolic pathway remodeling and adaptive responses. These results indicate that during the transition from RRS to GRS, amino acids and their derivatives not only act as substrates for protein biosynthesis but may also function as signaling molecules or precursors of flavor compounds. Previous studies have demonstrated that amino acid metabolism fluctuates widely during tomato fruit development and that Asp, Glu, and Met increase during on-vine ripening [11,50], while sulfur-containing amino acids such as methionine may further serve as precursors of volatile sulfur compounds contributing to fruit-specific flavor formation. Moreover, enzyme activity during fruit development remains relatively stable, whereas the levels of metabolites such as amino acids are more strongly affected by developmental stage [51], supporting the hypothesis that protein hydrolysis provides amino acids in the early stage, while in the later stage amino acids are transformed into functional compounds. Combined with previous reports on plant signaling peptides such as systemin, these findings suggest that tomato-derived peptides may also play potential roles in regulation and flavor formation.

### 3.3. Effects of Different Ripe Stages on Volatile Substances in Tomato Fruits

#### 3.3.1. Comparative Analysis of the Composition and Content of Volatile Substances in Tomato Fruits at Different Ripe Stages

**Figure 5:**
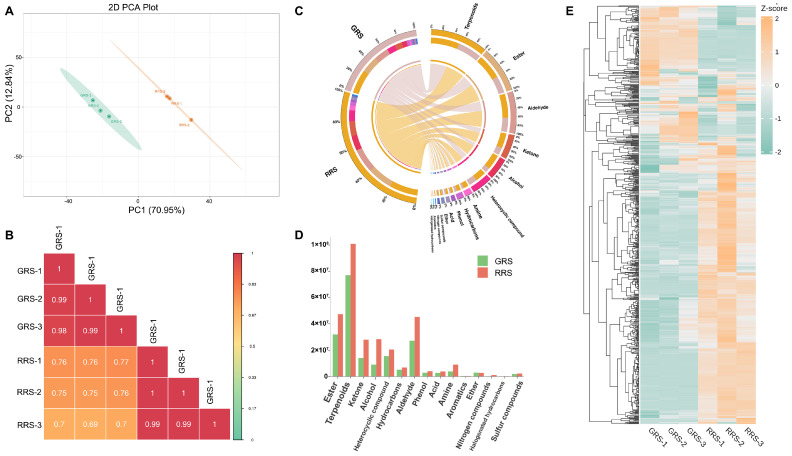
Analysis of volatile substances in tomato fruits. (**A**) PCA analysis of each group of samples. (**B**) Pearson product-moment correlation coefficients. (**C**) The proportion of volatile substances. (**D**) The relative content of volatile substances in different ripe stages. (**E**) Volatile substance content heatmap.

To gain a comprehensive understanding of the impact of different ripe stages on the fragrance of tomato fruits, a study was conducted all volatile metabolites were identified using a GC–MS detection platform and a custom database. Untargeted GC–MS profiling clearly discriminated the GRS and RRS samples (PCA: PC1 = 70.95%, PC2 = 12.04; Figure 5A). Biological replicates clustered tightly within each stage, whereas cross-stage correlations were lower (Figure 5B), indicating a strong stage effect. A total of 788 metabolites were detected, covering 15 chemical categories, including 159 esters, 156 terpenoids, 88 ketones, 68 alcohols, 67 heterocyclic compounds, 57 hydrocarbons, 52 aldehydes, 31 phenols, 28 acids, 21 amines, 21 aromatics, 17 ethers, 14 nitrogen compounds, 5 halogenated hydrocarbons, and 4 sulfur compounds (Figure 5C, Table S2). It is worth noting that esters, terpenoids and ketones together account for approximately half (51.14%) of the total identified volatile compounds, highlighting their significance in determining the aroma characteristics of tomato fruits. At different ripe stages, the composition and content of volatile substances in tomato fruits have changed, thereby altering the odor of the fruits. By comparing the differences in the relative content of various types of volatile substances (Figure 5D), it was found that the relative content of different substances showed a trend of “RRS > GRS”. The content of almost all types of compounds in RRS increased (except for aromatics and ethers). It is notable that the increments of ketone, alcohol, amine and nitrogen compounds were the largest. The heatmap analysis (Figure 5E) confirmed that most volatiles were more abundant in RRS, consistent with the stronger fruity–floral aroma of red-ripe fruits. To verify the robustness of the stage discrimination, PLS-DA analysis was performed, and the model was cross-validated through permutation testing (R^2^Y = 0.983, Q^2^ = 0.977), indicating reliable predictive performance and the absence of overfitting (Figure S2).

#### 3.3.2. The Influence of Different Maturity Stages on the Key Aroma-Active Compounds in Tomato Fruits

The rOAV results of key aroma-active compounds (ROAV ≥ 1) showed distinct class-dependent contributions to the overall flavor profile across maturity stages (Table 2). (5Z)-Octa-1,5-dien-3-one is a low-molecular-weight enone formed as a secondary product of polyunsaturated fatty-acid oxidation, with an exceptionally low odor threshold that confers intense geranium- and metallic-like notes at trace levels [52]. Multiple GC-olfactometry studies in tomato identify it among the most potent odorants, supporting its status as an impact compound in ripe-fruit aroma profiles. Its occurrence is consistent with autoxidative lipid-oxidation pathways active during ripening [53,54]. In our dataset, it exhibited the highest rOAV, increasing sharply from 11.91 in GRS to 100 in RRS, underscoring the dominant contribution of ketones to the intensified aroma of red-ripe fruit. β-Ionone is a C13 norisoprenoid produced by 9,10(9′,10′)-cleavage of β-carotene and is widely distributed in fruits, flowers, and vegetables [55]. Owing to its exceptionally low odor threshold, it confers intense woody, floral, and sweet notes at trace levels. Beyond flavor, β-ionone has garnered biomedical interest for reported anti-inflammatory, antibacterial, and anticancer activities [56]. In this study, its contribution increased markedly from GRS to RRS (rOAV 10.85 to 51.5), highlighting a pivotal role in shaping the floral–fruity character of red-ripe fruit. It is a cleavage product of β-carotene with woody, floral, and sweet notes, also displayed elevated contributions, rising from 10.85 in GRS to 51.5 in RRS, underscoring its pivotal role in the floral and fruity character of ripe fruit. In contrast, aldehydes such as (E)-2-nonenal and 2-nonenal, which are associated with fatty, green, and fresh odors, showed a declining trend from GRS (2.13 and 1.71, respectively) to RRS (1.65 and 1.32). This decrease suggests a weakening of grassy and green sensory attributes during ripening. Esters, including pentanoic acid, 3-methyl-, ethyl ester and butanoic acid, 3-methyl-, 2-phenylethyl ester, contributed fruity, sweet, and rose-like aromas. Their rOAV values were relatively stable between the two stages (1.41 to 1.30 and 0.82 to 1.19, respectively), indicating that esters provided consistent background sweetness and fruity tones throughout ripening. Notably, sulfur compounds also emerged as important contributors in the later stage. Dimethyl trisulfide, characterized by sulfurous, cooked onion, and fatty odors, increased from an rOAV of 0.53 in GRS to 1.20 in RRS, surpassing the threshold and enhancing the complexity of the ripe fruit aroma. Overall, these findings suggest that the transition from GRS to RRS involves a shift from green and fresh aldehydic notes to intensified floral, woody, and sulfurous characteristics. Such compositional changes not only define the distinctive aroma profile of red-ripe fruit but also contribute to its higher sensory appeal and greater commercial value compared with green-ripe fruit.

**Figure 6:**
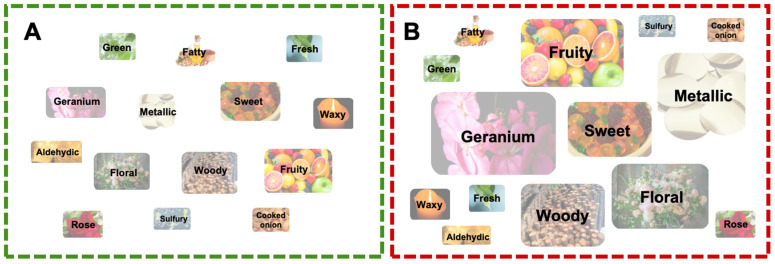
Chart showing flavor differences between green and red fruit. The size of each ellipse only indicates the strength of each type of aroma. Main flavors in (**A**) GRS and (**B**) RRS.

To more intuitively analyze the differences in the aroma characteristics of GRS and RRS, the critical volatile aroma compounds were classified and summarized according to aroma type. Figure 6 shows that the aroma characteristics of GRS and RRS can be described as being geranium and metallic (more prominent in RRS) followed by floral, woody, sweet, and fruity. RRS exhibited a more prominent sweet and fatty aroma. Moreover, fresh, rose, sulfury and cooked onion aromas contributed to the more intense aroma of RRS.

## 4. Conclusions

This study provides a comprehensive, stage-resolved characterization of metabolic and nutritional changes during cherry tomato ripening. By integrating targeted and untargeted metabolomic approaches, it identifies coordinated reprogramming of primary, specialized, and volatile metabolites that collectively define the transition from the green-ripe to the red-ripe stage. The results highlight how the accumulation of flavonols, phenolic acids, and antioxidant vitamins, together with the reduction in glycoalkaloids and the remodeling of aroma volatiles, contribute to the enhanced nutritional and sensory quality of red-ripe fruit. Beyond profiling individual compounds, this integrative framework establishes biochemical markers that can serve as indicators for fruit maturity and quality evaluation, and as potential metabolic targets for breeding programs aimed at improving antioxidant-related traits. Future work integrating transcriptomic and enzymatic analyses will help elucidate the underlying regulatory mechanisms and validate the physiological functions of the identified bioactive metabolites.

## Figures and Tables

**Table 1:** Total amino acid content of cherry tomato fruits at different ripe stages (mg/g FW).

Amino Acid	GRS (mg/g)	RRS (mg/g)
Aspartic acid (Asp)	0.887 ± 133 b	2.171 ± 0.220 a
Glutamic acid (Glu)	1.191± 0.191 b	11.619 ±0.924 a
Threonine (Thr)	7.113 ± 2.151 a	5.090 ± 0.647 a
Valine (Val)	0.176 ± 0.038 a	0.103 ± 0.004 b
Tyrosine (Tyr)	0.178 ± 0.049 a	0.143 ± 0.003 a
Isoleucine (Ile)	0.203 ± 0.035 a	0.121 ± 0.003 b
Leucine (Leu)	0.185 ± 0.023 a	0.217 ± 0.010 a
Arginine (Arg)	0.208 ± 0.059 b	0.331 ± 0.028 a
Cysteine (Cys)	0.124 ± 0.002 a	0.127 ± 0.001 a
Lysine (Lys)	0.234 ± 0.053 a	0.296 ± 0.018 a
Alanine (Ala)	0.222 ± 0.042 b	0.479 ± 0.032 a
Histidine (His)	0.176 ± 0.037 b	0.314 ± 0.020 a
Proline (Pro)	0.062 ± 0.011 b	0.157 ± 0.001 a
Methionine (Met)	0.031 ± 0.011 b	0.064 ± 0.011 a
Serine (Ser)	4.895 ± 1.150 b	6.034± 0.443 a
Glycine (Gly)	0.101 ± 0.004 a	0.103 ± 0.014 a
Phenylalanine (Phe)	0.565 ± 0.147 a	0.638 ± 0.069 a
Total	16.551 ± 4.083 b	28.006 ± 2.302 a

Note: Each value is the mean ± SE of three independent experiments. Different letters indicate significant differences between treatments (*p* < 0.05) according to Student’s t test.

**Table 2:** Key aroma active compounds with ROAV ≥ 1.

CAS	Compounds	Class	Odor	Threshold	ROAV	
					GRS	RRS
65767-22-8	(5Z)-Octa-1,5-dien-3-one	Ketone	geranium, metallic	0.000003	11.91	100
14901-07-6	β-Ionone	Terpenoids	floral, woody, sweet, fruity	0.000007	10.85	51.5
18829-56-6	2-Nonenal, (E)-	Aldehyde	fatty, green, fresh, aldehydic, fruity	0.00008	2.13	1.65
2463-53-8	2-Nonenal	Aldehyde	fatty, green, waxy, fresh, fruity	0.0001	1.71	1.32
5870-68-8	Pentanoic acid, 3-methyl-, ethyl ester	Ester	fruity	0.000008	1.41	1.30
140-26-1	Butanoic acid, 3-methyl-, 2-phenylethyl ester	Ester	floral, fruity, sweet, rose	0.00001	0.82	1.19
3658-80-8	Dimethyl trisulfide	Sulfur compounds	sulfury, cooked onion, fatty	0.000008	0.53	1.2

## Data Availability

The original contributions presented in this study are included in the article and Supplementary Materials. Further inquiries can be directed to the corresponding author.

## Supplementary Materials

The following supporting information can be downloaded at: https://www.mdpi.com/article/10.3390/antiox14111359/s1. Figure S1. OPLS-DA permutation test for non-volatile metabolite profiling. Figure S2. OPLS-DA permutation test for volatile metabolite profiling. Table S1. List of all identified non-volatile metabolites detected by UPLC–MS/MS. Table S2. List of all identified volatile compounds detected by HS-SPME–GC–MS. Table S3. QC validation, FDR corrections, and cross-validation statistical summary.
