# Supplementary material for: Dynamic Characterization of Antioxidant-Related, Non-Volatile, and Volatile Metabolite Profiles of Cherry Tomato During Ripening
Source: Antioxidants (Basel). 2025 Nov 13;14(11):1359. doi: 10.3390/antiox14111359 (PMC12649427; doi:10.3390/antiox14111359)
Supplement: Supplementary file 1 [file antioxidants-14-01359-s001.zip › supplementary figure 2.pdf]

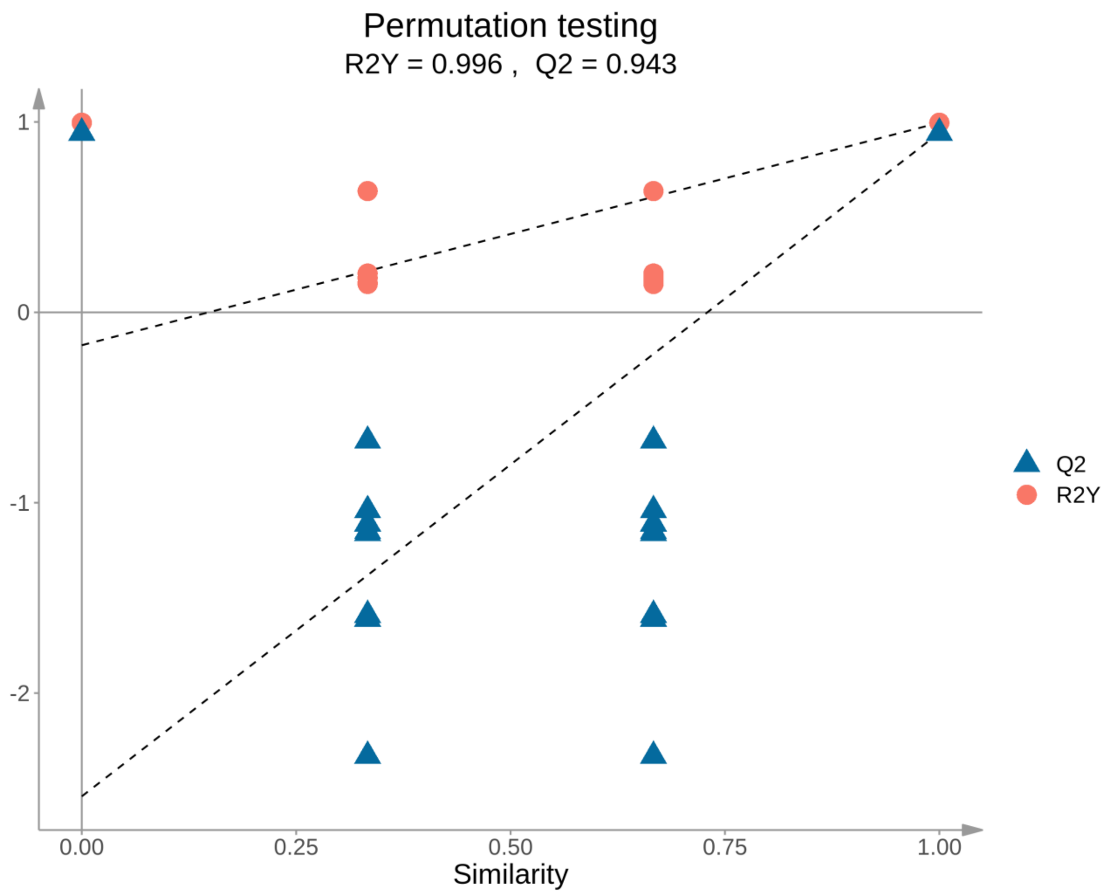

Figure S1. Composition analysis of different ripe stages on non-volatile substances in tomato fruits. OPLS-DA model validation plot.

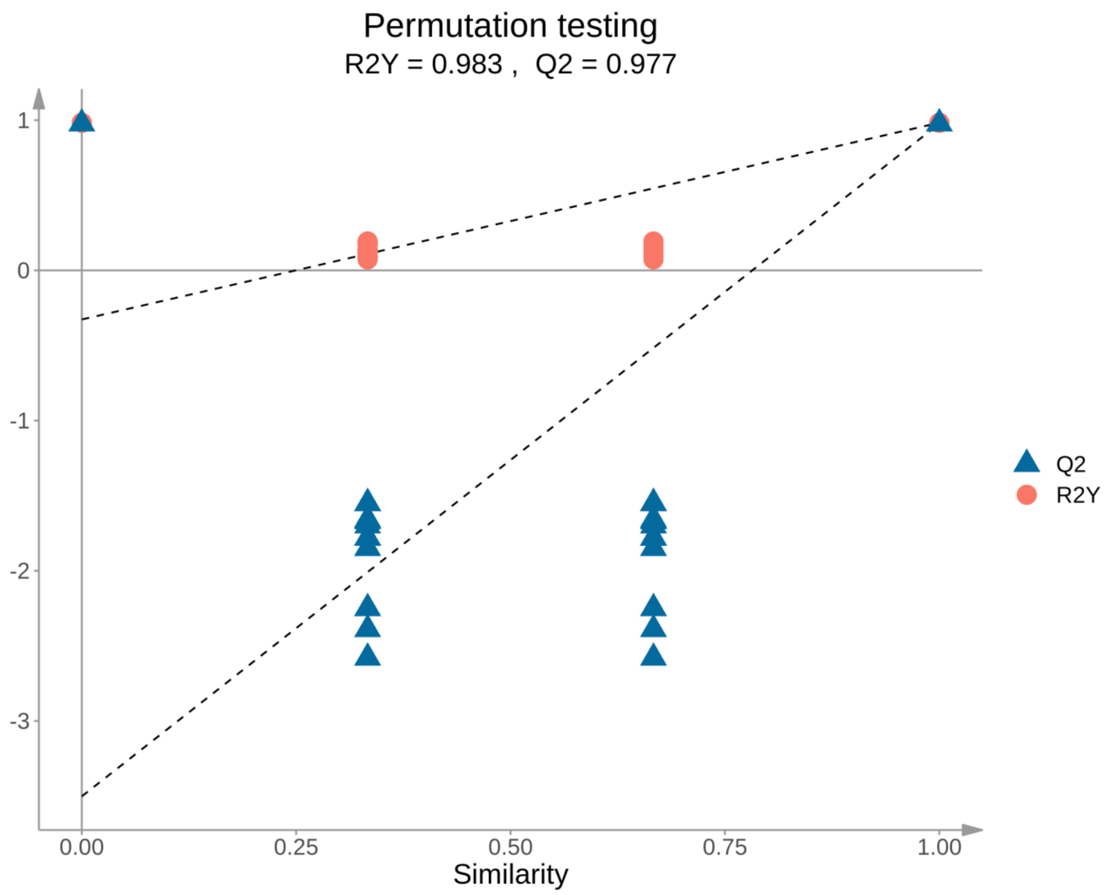

Figure S2. Composition analysis of different ripe stages on volatile substances in tomato fruits. OPLS-DA model validation plot.
